# Renoprotection by 5-Methoxytryptophan in Kidney Disease

**DOI:** 10.3390/biom16020223

**Published:** 2026-02-02

**Authors:** Jonah P. Gutierrez, Tram N. Diep, Shaona Niu, Liang-Jun Yan

**Affiliations:** Department of Pharmaceutical Sciences, UNT System College of Pharmacy, University of North Texas Health Science Center, Fort Worth, TX 76107, USA; jonahgutierrez@my.unthsc.edu (J.P.G.); tramdiep@my.unthsc.edu (T.N.D.); shaona.niu@unthsc.edu (S.N.)

**Keywords:** 5-methoxytryptophan (5-MTP), kidney, inflammation, fibrosis, mitophagy, oxidative damage

## Abstract

Kidney disease, be it acute or chronic, has a complex pathology and is a significant human health problem. Increasing interest has been focused on exploring therapeutic targets that can be used to safeguard kidney function under a variety of detrimental conditions. In this article, we review the protective effects of 5-methoxytryptophan (5-MTP), a tryptophan metabolite, on kidney injury. Published studies indicate that serum 5-MTP level is decreased in patients with chronic kidney disease (CKD), suggesting that 5-MTP is a biomarker for CKD and has therapeutic values. Indeed, rodent models of kidney injury induced by folic acid, lipopolysaccharide (LPS), unilateral ureteral obstruction (UUO), and ischemia/reperfusion all demonstrate that exogenous 5-MTP exhibits nephroprotective effects. The underlying mechanisms involve antioxidative damage via activating antioxidant systems such as heme oxygenase-1, anti-inflammation, anti-fibrosis, and enhanced mitophagy. To further explore the underlying mechanisms and the potential of 5-MTP as a kidney therapeutic compound, future studies need to include more rodent models of kidney injury induced by a variety of insults. Moreover, how to boost endogenous 5-MTP content and its potential synergistic effects with other therapeutic approaches aiming to combat kidney diseases also remain to be explored.

## 1. Introduction

The kidney is a vital organ for the maintenance of body function. It excretes water-soluble waste via glomerular filtration and selective tubular reabsorption of nutrients, minerals, and electrolytes [1]. As such, the kidney maintains blood pH within very narrow limits and regulates blood pressure. The kidney also produces and responds to hormones. Examples include erythropoietin production, vitamin D maturation, the aldosterone and parathyroid hormone, the renin–angiotensin system, and the vasopressin or antidiuretic hormone. The kidney can also participate in glucose production via the gluconeogenic pathway under anerobic conditions [2]. All these functions require a large amount of ATP produced by mitochondrial oxidative phosphorylation, a metabolic process that can also generate excessive reactive oxygen species (ROS) that can lead to oxidative damage to nephrons and result in kidney dysfunction [3,4]. Therefore, there is a constant need to search for therapeutic targets that can be used to safeguard kidney function under a variety of harmful conditions. In this review article, we focus on the protective role of 5-methoxytryptophan (5-MTP) in a variety of kidney diseases after a brief overview of kidney injuries such as acute kidney injury (AKI), chronic kidney disease (CKD), and diabetic kidney disease (DKD) as well as well-established underlying mechanisms such as oxidative stress, inflammation, and fibrosis.

### 1.1. Acute Kidney Injury

Clinically, AKI is a common renal disorder characterized by a sudden decline in kidney function and occurs more frequently in old-aged patients [5]. It is also highly prevalent in hospitalized individuals and is linked with numerous etiologies such as ischemia [6], cardiac surgery [7], sepsis [8], rhabdomyolysis [9], and drug or chemical toxicity [10]. AKI is often associated with increased mortality in severely ill patients. Moreover, even for patients who survive AKI, they could further develop CKD or end-stage renal failure [11]. Despite advances in our understanding of the underlying mechanisms of AKI, few therapeutic options are currently available.

### 1.2. Chronic Kidney Disease

CKD is characterized by a progressive decline of kidney function caused by various chronic disorders such as hypertension [12], environmental exposure to heavy metals [13,14], and diabetes [15]. It has been estimated that CKD may affect more than 10% of the Earth’s population. CKD can be identified by measuring a glomerular filtration rate (GFR) that is less than 60 mL/min/1.73 m^2^ for a period of more than 3 months while the albumin–creatinine ratio is constantly above 30 mg/g/day [16]. If unmanaged, CKD can become end-stage renal failure [17]. CKD is usually classified into five stages as shown in Table 1 and albuminuria categories are shown in Table 2.

### 1.3. Diabetic Kidney Disease

As an emerging global health issue, DKD is a major complication of diabetes mellitus. It is caused by persistent exposure of the kidney to hyperglycemia. DKD can develop in about 35% of diabetic patients [19]. It is a renal microvascular disorder of diabetes and is characterized by albuminuria levels greater than 30 mg/day accompanied by a declining GFR [20]. DKD can lead to anemia and vitamin D deficiency given that the kidney is responsible for erythropoietin generation and vitamin D maturation [4]. Redox imbalance, oxidative stress, inflammation, and fibrosis are all implicated in the pathogenesis of DKD [21].

### 1.4. Oxidative Stress

Oxidative stress occurs when cellular production of ROS overwhelms the cellular antioxidant defense system [22]. ROS can be produced by a variety of systems such as NADPH oxidases [23], the mitochondrial electron transport chain [24], dihydrolipoamide dehydrogenase [25], cyclooxygenase and lipoxygenase [26], and xanthine oxidase [27]. Excessive ROS can cause oxidative damage to proteins, lipids, and DNA, leading to dysfunction of the macromolecules [28]. On the other hand, disease status-caused functional decline of the antioxidant systems such as superoxide dismutase, catalase, glutathione peroxidase and reductase, thioredoxin reductase, and peroxiredoxin reductase can further perturb cellular redox balance toward aggravated oxidative stress. All these can collectively lead to the collapse of cellular function and cell death [29]. Table 3 lists major ROS-generating systems and antioxidant defense systems. With respect to the determination of oxidative stress levels, there are numerous methods that can be used. For example, protein oxidation can be measured as protein carbonyls [22], lipid peroxidation can be measured by malondialdehyde (MDA) using the assay for thiobarbituric acid reactive substances [30,31], and DNA damage can be measured as 8-oxo-deoxyguanosine [32,33]. Moreover, superoxide and hydrogen peroxide can also be measured by the use of a fluorescent probe called 2,7-dicholorofluorescin [34]. In the meantime, cellular antioxidant capacities such as glutathione levels [34], SOD, and catalase activities [35,36] can also be measured to indicate the magnitude of oxidative stress.

### 1.5. Inflammation

Renal inflammation, as a hallmark of and response to kidney injury [37], involves numerous signaling pathways. Participating cells in renal inflammation include neutrophils, monocytes, macrophages, T cells, B cells, and natural killer cells [38]. Inflammatory mediators can be classified into two family members. One is the chemokine family including SDF-1, CCL-2/MCP-1, and fractalkine; another is the cytokine family including TNF-α, IL-6, and IL-13 [38,39]. If unmanaged, renal inflammation can gradually lead to renal fibrosis [38,39].

### 1.6. Fibrosis

Renal fibrosis underlies AKI, CKD, and DKD as well as the AKI to CKD transition [40]. It is characterized by accumulation and excessive deposition of extracellular matrix (ECM). The fibrotic process also involves tubulointerstitial fibrosis, tubular atrophy, and glomerulosclerosis, which collectively can lead to renal failure [40]. Key signaling pathways involved in renal fibrosis include NF-kB, Nrf2, NADPH oxidase-4, autophagy activation, and transforming growth factor-β [41]. Additionally, oxidative stress and inflammation are highly involved in renal fibrosis [39], which has been repeatedly demonstrated and confirmed by the use of antioxidants and anti-inflammation molecules in a variety of animal models of kidney disease [42,43,44,45].

## 2. Tryptophan and Biosynthesis of 5-MTP

**Figure 1:**
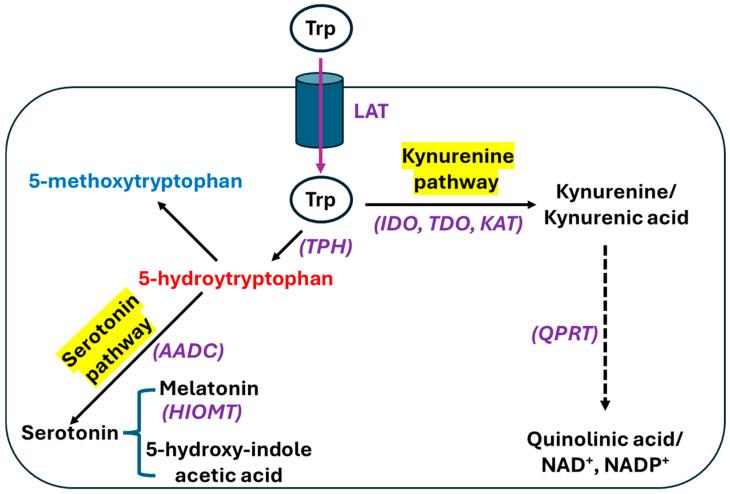
The catabolic pathways of tryptophan. Tryptophan enters cells via L-amino acid transporters (LATs) followed by two catabolic pathways: the serotonin pathway and the kynurenine pathway. 5-methoxytryptophan is synthesized from 5-hydroxytryptophan via two-step reactions as shown in Figure 2. LAT: L-amino acid transporter. The enzymes involved in the pathways are TPH, tryptophan hydroxylase; AADC, aromatic amino acid carboxylase; HIOMT, hydroxyindole O-methyltransferase; IDO, indoleamine 2,3-dioxygenase; TDO, tryptophan 2,3-dioxygenase; KAT, kynurenine amino transferase; and QPTR, quinolinate phosphoribosyltransferase.

**Figure 2:**
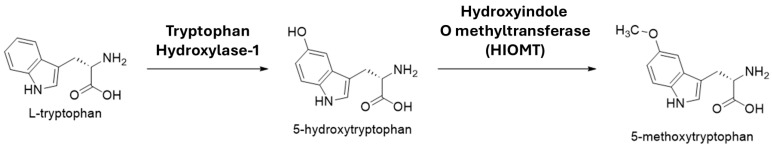
Synthesis of 5-MTP from 5-hydroxytryptophan. Note: the first step catalyzed by tryptophan hydroxylase is rate-limiting.

Tryptophan, as an essential amino acid, is both ketogenic and glucogenic in nature. It is involved in biosynthesis of proteins, lipids, glucose, and indole acetic acid [2]. Tryptophan is also the precursor of serotonin and melatonin [2,46]. It enters cells via L-amino acid transporters (LATs) and is then catabolized via two pathways (Figure 1) [47]. The catabolic pathway generating serotonin and melatonin is often called the serotonin pathway, while nearly 95% of ingested tryptophan is catabolized by a pathway called the kynurenine pathway, which produces NAD^+^/NADP^+^ and immunomodulatory molecules such as kynurenine and kynurenic acid [46,48,49]. It should be noted that de novo synthesis of NAD^+^/NADP^+^ from tryptophan can only occur in the liver and kidney while all the other tissues may use the salvage pathway as a source for NAD^+^/NADP^+^ [50]. As shown in Figure 2, tryptophan is also a source of 5-hydroxyindole acetic acid [51]. 5-MTP is synthesized via two reactions (Figure 2). In the first step, tryptophan is converted to 5-hydroxytryptophan (5-HTP) by tryptophan hydroxylase (TPH) [52]. This reaction is rate-limiting. In the second step, 5-HTP is converted to 5-MTP by hydroxyindole O-methyltransferase (HIOMT) [52]. 5-MTP, being a 5-methoxyindole metabolite [53,54], can also serve as a precursor for the synthesis of 5-methoxyindole-2-carboxylic acid (MICA), which has been rigorously evaluated by our laboratory for its neuroprotective role in ischemic stroke [55,56,57,58].

## 3. Potential Biological Functions of 5-MTP

5-MTP was originally named as cytoguardin due to its ability to suppress cyclooxygenase-2 (COX-2) in proliferating fibroblasts [59,60,61,62]. It was later confirmed that 5-MTP can also be made by other types of cells including smooth muscle cells, vascular endothelial cells, bronchial epithelial cells, and renal epithelial cells [63]. Moreover, bacteria in the gut microbiota can also produce 5-MTP [64]. Numerous studies have revealed that 5-MTP is a disease-fighting molecule as it can exhibit protective properties such as antioxidation, anti-inflammation, anti-fibrosis, anti-senescence, and anti-tumor properties [65,66,67,68,69]. In this review, we will focus on the protective effects of 5-MTP on kidney disease.

### 3.1. 5-MTP and Chronic Kidney Disease

Chen et al. first reported that 5-MTP content was lower in CKD patients than in healthy subjects [70]. Using UPLC-HDMS metabolomics and real-time PCR quantification techniques, the authors compared healthy individuals and CKD patients and analyzed the concentrations of 25 metabolites in serum. 5-MTP content showed an inverse correlation with kidney function, indicating that 5-MTP may be a biomarker of CKD. The same research group further discovered that serum 5-MTP had an anti-fibrotic effect in CKD [71]. These studies signify that exogenous 5-MTP has nephroprotective effects, which indeed have been explored and confirmed by other investigators, as discussed in this article.

Mogos et al. also found that serum 5-MTP level is an important biomarker for early-stage diabetic kidney disease [72]. The authors analyzed urine and serum amino acid metabolites in initial stages of DKD in type 2 diabetic patients. A total of 90 T2D patients were grouped into three subgroups based on their albuminuria stage from normal albuminuria to micro-albuminuria and macro-albuminuria, with each subgroup having 30 patients. Additionally, 20 healthy individuals were recruited as controls. Statistical analysis revealed that 5-MTP, among others, was more significant in terms of discrimination between the subgroups and the controls. This study indicates that 5-MTP not only can serve as a biomarker in early stage of DKD but also plays an active role in the progression of DKD. Nevertheless, whether 5-MTP is lower or higher in rodent models of DKD than in controls remains to be investigated.

### 3.2. Effect of 5-MTP on Lipopolysaccharide (LPS)-Induced Acute Kidney Injury

Sepsis caused by over-inflammatory response to infections can result in multiple organ failures including the kidney [73]. In animal models, septic AKI can be induced by a simple administration of LPS via intraperitoneal injection [74,75], which has been widely used to investigate septic mechanisms of kidney injury and also serves as a platform for testing the therapeutic values of numerous chemicals, compounds, and natural products [75]. In this septic kidney injury process, many inflammatory mediators such as interleukin-1, interleukin-6, and tumor necrosis factor-α can overload the kidneys and cause inflammation that further leads to tissue hypoxia and damage [76]. Using this LPS-induced AKI mouse model, Sun et al. demonstrated that exogenous 5-MTP exerts protective effects on kidney injury [77]. The authors further demonstrated that 5-MTP activated the Nrf2 transcription factor, leading to upregulation of heme oxygenase-1 (HO-1), which is a major antioxidant protein. This upregulation of the Nrf2 pathway also augmented renal tubular mitophagy reflected by LC3 immunohistochemical staining, thereby helping to maintain mitochondrial homeostasis and decrease mitochondrial oxidative stress. It should be noted that the authors’ interest in studying the effect of 5-MTP on septic AKI was triggered by their observation that serum 5-MTP levels in patients with AKI were significantly elevated when compared with control subjects, an observation seemingly in contrast with that of Chen et al. who reported that 5-MTP content in the serum of CKD patients was lower than in controls [71]. This difference might be due to the nature of the kidney injury in the respective study, that is, AKI vs. CKD. Nevertheless, it is possible that acute stress response in AKI may upregulate tryptophan metabolism, whereas the chronic response in CKD may exhaust tryptophan metabolism.

### 3.3. Effect of 5-MTP on Renal Ischemia/Reperfusion Injury

**Figure 3:**
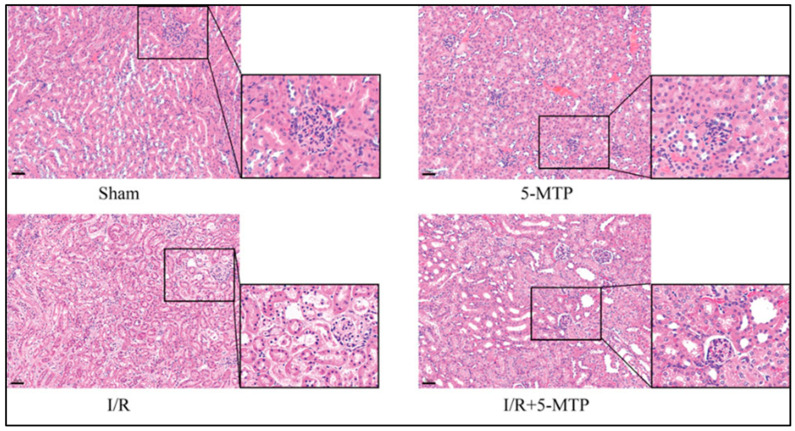
Hematoxylin–eosin staining of renal histopathological lesions induced by I/R and amelioration of the lesions by 5-MTP. This figure was reproduced from Li et al. [84]. Please refer to the methodological and statistical details as well as scale bar provided in the original reference.

Renal ischemia–reperfusion (I/R) injury can occur in many clinical settings such as cardiac surgery, systemic vasodilation, volume depletion, shock, decreased cardiac output, and kidney transplantation [78,79,80]. Decrease in renal blood flow often results in hypoxia and a lack of energy supply that culminate in cell death and kidney functional decline [81,82,83]. Using a mouse model of renal I/R injury, Li et al. tested the protective effects of 5-MTP in I/R kidney injury [84]. 5-MTP was administered to mice 30 min before ischemic surgery. The authors found that exogenous 5-MTP mitigated renal damage and improved kidney function. Hematoxylin–eosin staining clearly showed I/R-induced renal tissue damage that was ameliorated by 5-MTP pretreatment (Figure 3). The underlying mechanisms involved attenuated ER stress and apoptosis. Moreover, the Nrf2 pathway was also activated by 5-MTP with corresponding upregulation of HO-1. When Nrf2 knockout mice were used, the protective effects of 5-MTP were partially suppressed, demonstrating that 5-MTP’s protective effect on renal I/R injury is Nrf2-dependent.

### 3.4. Effects of 5-MTP on Renal Tissue Inflammation and Fibrosis

Unilateral ureteral obstruction (UUO) is a widely used animal model for induction of kidney inflammation and fibrosis [85,86,87]. Wu et al. used this established animal model to evaluate the effect of 5-MTP on UUO-induced kidney injury [88]. The authors treated mice with 5-MTP prior to UUO surgery and then measured toll-like receptor 2 (TLR2) function and TGF-β signaling pathways. The authors also used TLR2 knockout mice in their study. Results indicated that in wildtype mice, the protective effect of 5-MTP in the UUO model was due to the downregulation of TLR2 and was similar to that in the TLR2-deficient mice, demonstrating that 5-MTP protection against UUO kidney injury involving inflammation and fibrosis is mediated by TLR2. Moreover, renal fibrosis attenuated by 5-MTP was accompanied by decreased macrophage infiltration of the kidney tissue as reflected by decreased TGF-β levels. The authors concluded that 5-MTP is nephroprotective against UUO-induced renal fibrosis and inflammation by blocking TLR2 and TGF-β signaling pathways.

### 3.5. Effects of 5-MTP on CKD-Induced Cerebrovascular Injury

It is well-known that kidney disease has a negative impact on the brain [89,90]. Indeed, kidney–brain interactions have garnered increasing interest over the years [91,92]. While there are numerous studies on epidemiology and mechanisms regarding the kidney–brain axis [93,94], the role of 5-MTP in this axis remains unknown. To address this unmet need, Zhou et al. used an animal model of kidney injury induced by a high concentration of folic acid and then performed the Morris water maze test on these animals [95]. As expected, the kidneys were injured by folic acid as measured by blood urea nitrogen, and the CKD animals exhibited increased escape latency, indicating a cerebrovascular injury. Interestingly, the authors found that both kidney injury and brain injury were suppressed by 5-MTP, an effect that was partially reversed by NF-kB overexpression. This study demonstrates that CKD-induced cerebrovascular injury can be mitigated by 5-MTP via attenuating the NF-kB pathway.

### 3.6. Predictive Value of 5-MTP on Clinical Outcomes in Patients Having Septic AKI

As a potential biomarker of kidney injury, does 5-MTP have any predictive value in terms of clinical outcomes of patients with septic AKI? This question was addressed by Sun et al. [96]. The authors recruited 31 healthy individuals and 78 patients who were diagnosed with septic AKI. Serum 5-MTP contents were measured by targeted metabolomics and correlation between serum 5-MTP and kidney function was assessed. The authors found that serum 5-MTP was significantly increased in patients with septic AKI, and this increase was associated with blood urea nitrogen (BUN), serum creatinine, and eGFR values. Additionally, the authors also found that higher content of 5-MTP was linked to a faster recovery of renal function, and a lowered content of 5-MTP was linked to an increased 90-day mortality in septic AKI patients. Therefore, 5-MTP has a predictive value in the development of septic AKI and an early elevation in serum may positively influence the prognosis of septic AKI.

### 3.7. Does Microbial 5-MTP Have a Nephroprotective Effect?

The gut microbiota also produces 5-MTP [97,98]. Whether microbial 5-MTP can protect the kidney against a variety of kidney injuries has not been explicitly investigated. A recently published study by Gong et al. may shed light on this aspect [99]. The authors studied the role of microbial 5-MTP in the shedding of angiotensin-converting enzyme-2 (ACE2) from the intestinal epithelial cells and sepsis induced by gut leak. They found that ACE2 deficiency was clearly associated with a decrease in microbial production of 5-MTP. When septic mice were given exogenous 5-MTP, the gut leak was ameliorated via enhanced proliferation and repair of epithelial cells. This amelioration was mediated by the PI3-Akt-WEE1 signaling pathway. Therefore, it is conceivable that microbial 5-MTP can also exert a protective effect on kidney injury. In a separate study by Kuo et al. [64], it was found that microbial 5-MTP was low in patients undergoing hemodialysis, further indicating that microbial 5-MTP is involved in kidney function or dysfunction depending on the context of the kidney injury. Future studies need to be performed to evaluate under what disease conditions microbial 5-MTP is beneficial or detrimental.

### 3.8. Pharmacological Boosting of 5-MTP Levels for Renoprotection

Shenkang injection (SKI), a formula of traditional Chinese medicine, consisting of astragalus, rhubarb, and safflower, has been used to treat chronic kidney failure (CKF) induced by adenine in rats. Zhou et al. [100] investigated the protective mechanisms of SKI and found that when CKF rats were given SKI, renal content of 5-MTP was significantly elevated when compared with CKF rats that were not treated with SKI. Moreover, SKI also attenuated the PI3/Akt and NF-kB signaling pathways. This study strongly suggests that elevation of 5-MTP is one of the mechanisms by which SKI ameliorates CKF. Nonetheless, whether the elevation of 5-MTP is due to increased enzymatic activity of tryptophan hydroxylase remains unknown. This study also suggests that boosting 5-MTP content by pharmacological agents, dietary modulation, or drugs is possible for nephroprotective purposes.

## 4. Future Perspectives

A PubMed search using the keywords “kidney” and “methoxytryptophan” yielded only 14 articles, which were the basis of this review article. This PubMed search outcome also indicates that more studies need to be performed to widen and deepen our knowledge on the protective effects and mechanisms of 5-MTP on kidney injuries. In our opinion, numerous rodent models of AKI and CKD remain to be explored. These models are listed in Table 4. Table 4 also gives potential approaches that may facilitate or enhance endogenous production of 5-MTP for renoprotective purposes. For example, it would be interesting to know whether caloric restriction including methionine restriction [101] and isoleucine restriction [102] or ketogenic diet can promote 5-MTP production in the context of kidney injury. Targeting delivery of 5-MTP to the kidneys using nanoparticle delivery techniques [103,104,105] is also worth exploring.

Finally, it should also be emphasized that given that 5-MTP acts as an antioxidant that attenuates renal oxidative stress, inflammation, and fibrosis, the underlying mechanisms remain to be comprehensively elucidated. One area would be investigation of the relationship between 5-MTP and NAD^+^/NADH redox balance. Does 5-MTP elevate NAD^+^ levels? If so, how is the NAD^+^-dependent network modulated by this elevation? This would require studies on NAD^+^-dependent enzymes including pyruvate dehydrogenase complex, mitochondrial complex I, NAD kinase, sirtuins, poly-(ADP)-ribosylase, and CD38 [10,106]. It is likely that 5-MTP may boost NAD^+^ content and ameliorate kidney injury caused by a variety of endogenous and exogenous insults. Another area would be the effects of 5-MTP on the infiltration of neutrophils and macrophages into the kidney upon injury. Indeed, how 5-MTP affects NETosis [107] and macrophage release of various inflammatory cytokines such as TNF-α and IL-6 [108] should be investigated.

**Table 4:** Rodent models that remain to be evaluated for the renoprotective effects of 5-MTP.

Model	References
Glycerol-induced kidney injury	[109,110]
5/6 Nephrectomy-induced kidney injury	[111,112]
Doxorubicin-induced kidney injury	[113]
Cisplatin-induced kidney injury	[10]
Cadmium- and other metal-induced kidney injury	[114,115,116]
Adenine-induced CKD	[117,118]
Streptozotocin-induced diabetic kidney disease (DKD)	[119]
Zucker diabetic rats, ZSF-1 rats, db/db mice	[120,121,122]
High-fat diet/streptozotocin-induced DKD	[123,124,125]
High fructose/streptozotocin	[126,127]
Nicotinamide/streptozotocin-induced DKD	[15]
D-galactose-induced kidney injury	[128,129,130]
Cecal ligation and puncture-induced kidney injury	[131,132]
Caloric restriction	[133,134,135]
Ketogenic diet and ketone body ingestion	[136,137,138]

## 5. Summary

**Figure 4:**
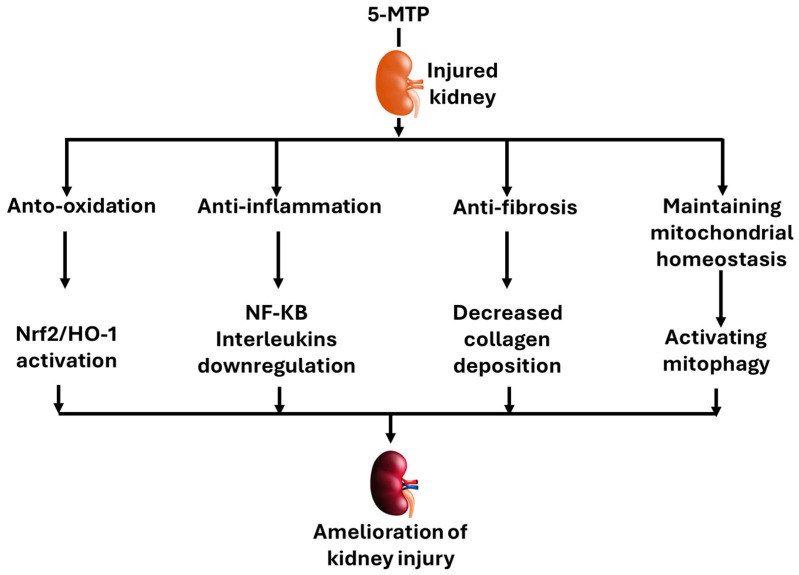
Mechanisms by which 5-MTP protects against kidney injury discussed in this review. 5-MTP possesses protective properties such as antioxidation, anti-inflammation, anti-fibrosis, and maintenance of mitochondrial homeostasis. Mechanisms for each property are also shown such as Nrf2 activation, NF-kB downregulation, decreased collagen accumulation, and mitophagy activation.

In this article, we reviewed the protective effects of 5-MTP on kidney injury in various models shown in Table 5. We started with a summary of findings in humans that serum 5-MTP was decreased in CKD patients. This was followed by discussions of the nephroprotective effects of 5-MTP in animal models of AKI or CKD. The rodent models covered include kidney injury induced by folic acid, LPS, UUO, and ischemia–reperfusion. The mechanisms by which 5-MTP protects against kidney injury involve upregulation of antioxidant defense systems, downregulation of oxidative damage, decreased fibrosis, and increased mitophagy (Figure 4). Overall, studies discussed in this article indicate that 5-MTP is not only a potential biomarker in kidney injury but also a renal protectant. Nevertheless, in addition to the above-mentioned potential studies that remain to be performed, future studies regarding endogenous boosting of 5-MTP and its synergistic effects with other therapeutic approaches also remain to be conducted.

## Figures and Tables

**Table 1:** Classification of CKD stages.

Stage	GFR Value *	Classification
1.	>90	Normal and healthy
2.	60–89	Slightly decreased renal function
3A.	45–59	Mild to moderately decreased renal function
3B.	30–44	Moderate to severe renal dysfunction
4.	15–29	Severely decreased renal function
5.	<15	Renal failure

* The unit for the GFR value is mL/min/1.73 m^2^. This table was reproduced from reference [18].

**Table 2:** Albuminuria categories. The ratio between albumin and creatinine (A/C) in urine samples is shown.

Category	24 h Albuminuria (mg/24 h)	A/C Ratio (mg/g)	Classification
A1	<30	<30	Normal to discrete
A2	30–300	30–300	Moderate
A3	>300	>300	Severe

This table was reproduced from reference [18].

**Table 3:** Major cellular ROS-generating systems and antioxidant defense systems.

ROS Generation Systems	Antioxidant Defense Systems
NADPH oxidases	Superoxide dismutase
Electron transport chain	Catalase
Dihydrolipoamide dehydrogenase	Glutathione (GSH)
Xanthine oxidase	GSH reductase and GSH peroxidase
Cyclooxygenase	Thioredoxin reductase
Lipoxygenase	Peroxiredoxin reductase
Cytochrome P450	Vitamin C and E, CoQ-10, etc.
Peroxynitrite produced by superoxide/nitric oxide	Nrf2/HO-1/NQO1

**Table 5:** Kidney injury models covered in this article.

	Models	References
1.	AKI in humans	[96]
2.	CKD in humans	[70,71]
3.	Rodent model of LPS-induced kidney injury	[77]
4.	Rodent model of folic acid-induced kidney injury	[139]
5.	Rodent model of ischemia/reperfusion injury	[84]
6.	Unilateral ureteral obstruction (UUO) animal model	[88]
7.	Early stage of DKD in humans	[72]

## Data Availability

No new data were created or analyzed in this study. Data sharing is not applicable to this article.

## References

[1] Reed S. (2009). Essential Physiological Biochemistry: An Organ-Based Approach.

[2] Lieberman M., Peet A. (2023). Marks’ Basic Medical Biochemistry: A Clinical Approach.

[3] Duann P., Lin P.H. (2017). Mitochondria Damage and Kidney Disease. Adv. Exp. Med. Biol..

[4] Kamt S.F., Liu J., Yan L.J. (2023). Renal-Protective Roles of Lipoic Acid in Kidney Disease. Nutrients.

[5] Marquez-Exposito L., Tejedor-Santamaria L., Santos-Sanchez L., Valentijn F.A., Cantero-Navarro E., Rayego-Mateos S., Rodrigues-Diez R.R., Tejera-Munoz A., Marchant V., Sanz A.B. (2021). Acute Kidney Injury is Aggravated in Aged Mice by the Exacerbation of Proinflammatory Processes. Front. Pharmacol..

[6] Yan Y., Bai J., Zhou X., Tang J., Jiang C., Tolbert E., Bayliss G., Gong R., Zhao T.C., Zhuang S. (2015). P2X7 receptor inhibition protects against ischemic acute kidney injury in mice. Am. J. Physiol. Cell Physiol..

[7] Rosner M.H., Okusa M.D. (2006). Acute kidney injury associated with cardiac surgery. Clin. J. Am. Soc. Nephrol..

[8] Wang Y., Zhu J., Liu Z., Shu S., Fu Y., Liu Y., Cai J., Tang C., Liu Y., Yin X. (2021). The PINK1/PARK2/optineurin pathway of mitophagy is activated for protection in septic acute kidney injury. Redox Biol..

[9] Zhang Z., Li J., Chen S., Peng J., Luo X., Wang L., Liao R., Zhao Y., Zhang S., Su B. (2025). Genetic and Pharmacological Inhibition of NOX4 Protects Against Rhabdomyolysis-Induced Acute Kidney Injury Through Suppression of Endoplasmic Reticulum Stress. Antioxidants.

[10] Iskander A., Yan L.J. (2022). Cisplatin-Induced Kidney Toxicity: Potential Roles of Major NAD(+)-Dependent Enzymes and Plant-Derived Natural Products. Biomolecules.

[11] Fiorentino M., Grandaliano G., Gesualdo L., Castellano G. (2018). Acute Kidney Injury to Chronic Kidney Disease Transition. Contrib. Nephrol..

[12] Ameer O.Z. (2022). Hypertension in chronic kidney disease: What lies behind the scene. Front. Pharmacol..

[13] Robles-Osorio M.L., Sabath-Silva E., Sabath E. (2015). Arsenic-mediated nephrotoxicity. Ren. Fail..

[14] Zhang L., Han T., Qian R., Xiao Y., Zhu W., Xie L. (2026). Effect and mechanism of Ganoderma leucocontextum extract on cadmium-toxic nephropathy. J. Ethnopharmacol..

[15] Yan L.J. (2022). The Nicotinamide/Streptozotocin Rodent Model of Type 2 Diabetes: Renal Pathophysiology and Redox Imbalance Features. Biomolecules.

[16] Pedraza-Chaverri J., Eugenio-Pérez D., Medina-Fernández L.Y., Saldivar-Anaya J.A., Molina-Jijón E., Ahmad R. (2016). Role of Dietary Antioxidant Agents in Chronic Kidney Disease. Free Radicals and Diseases.

[17] Hsu C.Y. (2007). Linking the population epidemiology of acute renal failure, chronic kidney disease and end-stage renal disease. Curr. Opin. Nephrol. Hypertens..

[18] Ammirati A.L. (2020). Chronic Kidney Disease. Rev. Assoc. Médica Bras..

[19] Diep T.N., Liu H., Yan L.-J. (2025). Beneficial Effects of Butyrate on Kidney Disease. Nutrients.

[20] Beernink J.M., van Mil D., Laverman G.D., Heerspink H.J.L., Gansevoort R.T. (2025). Developments in albuminuria testing: A key biomarker for detection, prognosis and surveillance of kidney and cardiovascular disease-A practical update for clinicians. Diabetes Obes. Metab..

[21] Yan L.J. (2021). NADH/NAD(+) Redox Imbalance and Diabetic Kidney Disease. Biomolecules.

[22] Yan L.J. (2009). Analysis of oxidative modification of proteins. Curr. Protoc. Protein Sci..

[23] Kuroda J., Sadoshima J. (2010). NADPH oxidase and cardiac failure. J. Cardiovasc. Transl. Res..

[24] Wu J., Jin Z., Yan L.J. (2017). Redox imbalance and mitochondrial abnormalities in the diabetic lung. Redox Biol..

[25] Yang X., Song J., Yan L.J. (2019). Chronic Inhibition of Mitochondrial Dihydrolipoamide Dehydrogenase (DLDH) as an Approach to Managing Diabetic Oxidative Stress. Antioxidants.

[26] Andres C.M.C., de la Lastra J.M.P., Juan C.A., Plou F.J., Perez-Lebena E. (2022). Hypochlorous Acid Chemistry in Mammalian Cells-Influence on Infection and Role in Various Pathologies. Int. J. Mol. Sci..

[27] Nishino T., Okamoto K., Eger B.T., Pai E.F. (2008). Mammalian xanthine oxidoreductase—Mechanism of transition from xanthine dehydrogenase to xanthine oxidase. FEBS J..

[28] Ames B.N., Shigenaga M.K. (1992). Oxidants are a major contributor to aging. Ann. N. Y. Acad. Sci..

[29] Miwa S., Muller F.L., Beckman K.B., Miwa S., Beckman K.B., Muller F.L. (2008). The basics of oxidative biochemistry. Oxidative Stress in Aging.

[30] Yan L.J., Lodge J.K., Traber M.G., Packer L. (1997). Apolipoprotein B carbonyl formation is enhanced by lipid peroxidation during copper-mediated oxidation of human low-density lipoproteins. Arch. Biochem. Biophys..

[31] Yan L.J., Lodge J.K., Traber M.G., Matsugo S., Packer L. (1997). Comparison between copper-mediated and hypochlorite-mediated modifications of human low density lipoproteins evaluated by protein carbonyl formation. J. Lipid Res..

[32] Yegin S.C., Dede S., Mis L., Yur F. (2017). Effects of Zinc Supplementation on DNA Damage in Rats with Experimental Kidney Deficiency. Biol. Trace Elem. Res..

[33] Baek J.H., Yalamanoglu A., Brown R.P., Saylor D.M., Malinauskas R.A., Buehler P.W. (2018). Renal Toxicodynamic Effects of Extracellular Hemoglobin After Acute Exposure. Toxicol. Sci..

[34] Yan L.J., Rajasekaran N.S., Sathyanarayanan S., Benjamin I.J. (2005). Mouse HSF1 disruption perturbs redox state and increases mitochondrial oxidative stress in kidney. Antioxid. Redox Signal..

[35] Yan W., Xu Y., Yuan Y., Tian L., Wang Q., Xie Y., Shao X., Zhang M., Ni Z., Mou S. (2017). Renoprotective mechanisms of Astragaloside IV in cisplatin-induced acute kidney injury. Free Radic. Res..

[36] Sedaghat Z., Kadkhodaee M., Seifi B., Salehi E., Najafi A., Dargahi L. (2013). Remote preconditioning reduces oxidative stress, downregulates cyclo-oxygenase-2 expression and attenuates ischaemia-reperfusion-induced acute kidney injury. Clin. Exp. Pharmacol. Physiol..

[37] Menini S., Iacobini C., Vitale M., Pugliese G. (2020). The Inflammasome in Chronic Complications of Diabetes and Related Metabolic Disorders. Cells.

[38] Black L.M., Lever J.M., Agarwal A. (2019). Renal Inflammation and Fibrosis: A Double-edged Sword. J. Histochem. Cytochem..

[39] Luo Y., Long M., Wu X., Zeng L. (2025). Targeting the NLRP3 inflammasome in kidney disease: Molecular mechanisms, pathogenic roles, and emerging small-molecule therapeutics. Front. Immunol..

[40] Liu H., Xiang X., Shi C., Guo J., Ran T., Lin J., Dong F., Yang J., Miao H. (2025). Oxidative stress and inflammation in renal fibrosis: Novel molecular mechanisms and therapeutic targets. Chem. Biol. Interact..

[41] Chen X.C., Huang L.F., Tang J.X., Wu D., An N., Ye Z.N., Lan H.Y., Liu H.F., Yang C. (2023). Asiatic acid alleviates cisplatin-induced renal fibrosis in tumor-bearing mice by improving the TFEB-mediated autophagy-lysosome pathway. Biomed. Pharmacother..

[42] Jiao H., Zhang M., Chen L., Zhang Z. (2025). Traditional Chinese Medicine targeting the TGF-beta/Smad signaling pathway as a potential therapeutic strategy for renal fibrosis. Front. Pharmacol..

[43] Zeng Z., Lao B., Liu K., Cao Y., Liao Y., Zhou J. (2025). Review of the Delay of Chronic Kidney Disease Progression by Wuling San on Improving Renal Fibrosis. Basic Clin. Pharmacol. Toxicol..

[44] Fu Y., Tang C., Cai J., Chen G., Zhang D., Dong Z. (2018). Rodent models of AKI-CKD transition. Am. J. Physiol. Renal Physiol..

[45] Nogueira A., Pires M.J., Oliveira P.A. (2017). Pathophysiological Mechanisms of Renal Fibrosis: A Review of Animal Models and Therapeutic Strategies. In Vivo.

[46] Tsuji A., Ikeda Y., Yoshikawa S., Taniguchi K., Sawamura H., Morikawa S., Nakashima M., Asai T., Matsuda S. (2023). The Tryptophan and Kynurenine Pathway Involved in the Development of Immune-Related Diseases. Int. J. Mol. Sci..

[47] Ramprasath T., Han Y.M., Zhang D., Yu C.J., Zou M.H. (2021). Tryptophan Catabolism and Inflammation: A Novel Therapeutic Target For Aortic Diseases. Front. Immunol..

[48] Gagliardi F., De Domenico P., Snider S., Roncelli F., Comai S., Mortini P. (2025). Immunomodulatory mechanisms driving tumor escape in glioblastoma: The central role of IDO and tryptophan metabolism in local and systemic immunotolerance. Crit. Rev. Oncol. Hematol..

[49] Umeda R., Ito Y., Minatoguchi S., Koide S., Takahashi K., Hayashi H., Hasegawa M., Yuzawa Y., Yamamoto Y., Saito K. (2025). Investigation of the Impact of Tryptophan-Metabolizing Enzymes and Kynurenic Acid on Antibody-Mediated Glomerulonephritis. FASEB J..

[50] Liu L., Su X., Quinn W.J., Hui S., Krukenberg K., Frederick D.W., Redpath P., Zhan L., Chellappa K., White E. (2018). Quantitative Analysis of NAD Synthesis-Breakdown Fluxes. Cell Metab..

[51] Satyanarayana U., Chakrapani U. (2020). Biochemistry.

[52] Fang L., Chen H., Kong R., Que J. (2020). Endogenous tryptophan metabolite 5-Methoxytryptophan inhibits pulmonary fibrosis by downregulating the TGF-beta/SMAD3 and PI3K/AKT signaling pathway. Life Sci..

[53] Morton D.J. (1987). Production of methoxyindoles in vitro from methoxytryptophan by rat pineal gland. J. Pineal Res..

[54] Su Y.C., Wang C.C., Weng J.H., Yeh S.A., Chen P.J., Hwang T.Z., Chen H.C. (2022). 5-Methoxytryptophan Sensitizing Head and Neck Squamous Carcinoma Cell to Cisplatitn Through Inhibiting Signal Transducer and Activator of Transcription 3 (STAT3). Front. Oncol..

[55] Yan L.J., Wang Y. (2023). Roles of Dihydrolipoamide Dehydrogenase in Health and Disease. Antioxid. Redox Signal..

[56] Sumien N., Huang R., Chen Z., Vann P.H., Wong J.M., Li W., Yang S., Forster M.J., Yan L.J. (2020). Effects of dietary 5-methoxyindole-2-carboxylic acid on brain functional recovery after ischemic stroke. Behav. Brain Res..

[57] Wu J., Jin Z., Yang X., Yan L.J. (2018). Post-ischemic administration of 5-methoxyindole-2-carboxylic acid at the onset of reperfusion affords neuroprotection against stroke injury by preserving mitochondrial function and attenuating oxidative stress. Biochem. Biophys. Res. Commun..

[58] Wu J., Li R., Li W., Ren M., Thangthaeng N., Sumien N., Liu R., Yang S., Simpkins J.W., Forster M.J. (2017). Administration of 5-methoxyindole-2-carboxylic acid that potentially targets mitochondrial dihydrolipoamide dehydrogenase confers cerebral preconditioning against ischemic stroke injury. Free Radic. Biol. Med..

[59] Cheng H.H., Kuo C.C., Yan J.L., Chen H.L., Lin W.C., Wang K.H., Tsai K.K., Guven H., Flaberg E., Szekely L. (2012). Control of cyclooxygenase-2 expression and tumorigenesis by endogenous 5-methoxytryptophan. Proc. Natl. Acad. Sci. USA.

[60] Wu K.K., Cheng H.H., Chang T.C. (2014). 5-methoxyindole metabolites of L-tryptophan: Control of COX-2 expression, inflammation and tumorigenesis. J. Biomed. Sci..

[61] Deng W.G., Saunders M., Gilroy D., He X.Z., Yeh H., Zhu Y., Shtivelband M.I., Ruan K.H., Wu K.K. (2002). Purification and characterization of a cyclooxygenase-2 and angiogenesis suppressing factor produced by human fibroblasts. FASEB J..

[62] Wu K.K. (2021). Cytoguardin: A Tryptophan Metabolite against Cancer Growth and Metastasis. Int. J. Mol. Sci..

[63] You L.L., Luo X.B., Zhou W.Q., Zhang R.C., Li Z.H., Xu J.X., Ran J., Xu J. (2025). Aerobic exercise modulates aortic chondrogenesis and calcification via 5-methoxytryptophan and P38MAPK in atherosclerotic rats. Exp. Gerontol..

[64] Kuo T.H., Wu P.H., Liu P.Y., Chuang Y.S., Tai C.J., Kuo M.C., Chiu Y.W., Lin Y.T. (2025). Identification of Gut Microbiome Signatures Associated with Serotonin Pathway in Tryptophan Metabolism of Patients Undergoing Hemodialysis. Int. J. Mol. Sci..

[65] Lee C.M., Chien T.R., Wang J.S., Chen Y.W., Chen C.Y., Kuo C.C., Chiang L.T., Wu K.K., Hsu W.T. (2025). 5-Methoxytryptophan attenuates oxidative stress-induced downregulation of PINK1 and mitigates mitochondrial damage and apoptosis in cardiac myocytes. Free Radic. Biol. Med..

[66] Lai X., Wan X., Bao X., Yang Q., Zhang W., Tan Y., Cai X., Wang Z., Li Y., Liu C. (2025). 5-Methoxytryptophan attenuates hypobaric hypoxia induced acute lung injury by alleviating lipid peroxidation via targeting peroxiredoxin 6. Redox Biol..

[67] Wu K.K., Kuo C.C., Yet S.F., Lee C.M., Liou J.Y. (2020). 5-methoxytryptophan: An arsenal against vascular injury and inflammation. J. Biomed. Sci..

[68] Wu K.K. (2021). Control of Mesenchymal Stromal Cell Senescence by Tryptophan Metabolites. Int. J. Mol. Sci..

[69] Ho Y.C., Wu M.L., Su C.H., Chen C.H., Ho H.H., Lee G.L., Lin W.S., Lin W.Y., Hsu Y.J., Kuo C.C. (2016). A Novel Protective Function of 5-Methoxytryptophan in Vascular Injury. Sci. Rep..

[70] Chen D.Q., Cao G., Chen H., Liu D., Su W., Yu X.Y., Vaziri N.D., Liu X.H., Bai X., Zhang L. (2017). Gene and protein expressions and metabolomics exhibit activated redox signaling and wnt/beta-catenin pathway are associated with metabolite dysfunction in patients with chronic kidney disease. Redox Biol..

[71] Chen D.Q., Cao G., Chen H., Argyopoulos C.P., Yu H., Su W., Chen L., Samuels D.C., Zhuang S., Bayliss G.P. (2019). Identification of serum metabolites associating with chronic kidney disease progression and anti-fibrotic effect of 5-methoxytryptophan. Nat. Commun..

[72] Mogos M., Socaciu C., Socaciu A.I., Vlad A., Gadalean F., Bob F., Milas O., Cretu O.M., Suteanu-Simulescu A., Glavan M. (2023). Metabolomic Investigation of Blood and Urinary Amino Acids and Derivatives in Patients with Type 2 Diabetes Mellitus and Early Diabetic Kidney Disease. Biomedicines.

[73] Peerapornratana S., Manrique-Caballero C.L., Gomez H., Kellum J.A. (2019). Acute kidney injury from sepsis: Current concepts, epidemiology, pathophysiology, prevention and treatment. Kidney Int..

[74] Singh A.P., Junemann A., Muthuraman A., Jaggi A.S., Singh N., Grover K., Dhawan R. (2012). Animal models of acute renal failure. Pharmacol. Rep..

[75] Doi K., Leelahavanichkul A., Yuen P.S., Star R.A. (2009). Animal models of sepsis and sepsis-induced kidney injury. J. Clin. Investig..

[76] Goncalves G.M., Zamboni D.S., Camara N.O. (2010). The role of innate immunity in septic acute kidney injuries. Shock.

[77] Sun X., Wang H., Liu Y., Yang Y., Wang Y., Liu Y., Ai S., Shan Z., Luo P. (2024). 5-Methoxytryptophan Alleviates Lipopolysaccharide-Induced Acute Kidney Injury by Regulating Nrf2-Mediated Mitophagy. J. Inflamm. Res..

[78] Liu H., Diep T.N., Yan L.-J., Wang Y.-X. (2025). Mitigating Oxidative Stress and Inflammation: The Protective Role of β-lapachone in Kidney Disease. Inflammation: From Chemical Mediators and Pathophysiology to Practice and Treatment.

[79] Smith T., Zaidi A., Brown C.V.M., Pino-Chavez G., Bowen T., Meran S., Fraser D., Chavez R., Khalid U. (2024). Robust Rat and Mouse Models of Bilateral Renal Ischemia Reperfusion Injury. In Vivo.

[80] Fu Y., Xiang Y., Wei Q., Ilatovskaya D., Dong Z. (2024). Rodent models of AKI and AKI-CKD transition: An update in 2024. Am. J. Physiol. Renal Physiol..

[81] Zhuang S., Lu B., Daubert R.A., Chavin K.D., Wang L., Schnellmann R.G. (2009). Suramin promotes recovery from renal ischemia/reperfusion injury in mice. Kidney Int..

[82] Zhu Y.B., Zhang Y.P., Zhang J., Zhang Y.B. (2016). Evaluation of Vitamin C Supplementation on Kidney Function and Vascular Reactivity Following Renal Ischemic Injury in Mice. Kidney Blood Press. Res..

[83] Wu L., Li Q., Liu S., An X., Huang Z., Zhang B., Yuan Y., Xing C. (2019). Protective effect of hyperoside against renal ischemia-reperfusion injury via modulating mitochondrial fission, oxidative stress, and apoptosis. Free Radic. Res..

[84] Li S., Yang H., Zhang B., Li L., Li X. (2025). 5-methoxytryptophan ameliorates renal ischemia/reperfusion injury by alleviating endoplasmic reticulum stress-mediated apoptosis through the Nrf2/HO-1 pathway. Front. Pharmacol..

[85] Li X., Ma T.K., Wang P., Shi H., Hai S., Qin Y., Zou Y., Zhu W.T., Li H.M., Li Y.N. (2024). HOXD10 attenuates renal fibrosis by inhibiting NOX4-induced ferroptosis. Cell Death Dis..

[86] Su C.T., Jao T.M., Urban Z., Huang Y.J., See D.H.W., Tsai Y.C., Lin W.C., Huang J.W. (2021). LTBP4 affects renal fibrosis by influencing angiogenesis and altering mitochondrial structure. Cell Death Dis..

[87] Miguel V., Ramos R., Garcia-Bermejo L., Rodriguez-Puyol D., Lamas S. (2021). The program of renal fibrogenesis is controlled by microRNAs regulating oxidative metabolism. Redox Biol..

[88] Wu J.Y., Lee G.L., Chueh Y.F., Kuo C.C., Hsu Y.J., Wu K.K. (2025). 5-Methoxytryptophan Protects against Toll-Like Receptor 2-Mediated Renal Tissue Inflammation and Fibrosis in a Murine Unilateral Ureteral Obstruction Model. J. Innate Immun..

[89] Chen J., Fu L., Li M., Xie K., Li X., Zhou X.J., Yang L., Zhang L., Xue C., Mao Z. (2025). Decreased brain-derived neurotrophic factor expression in chronic kidney disease: Integrated clinical and experimental evidence. Front. Mol. Biosci..

[90] Ito H., Mori T. (2025). The brain-kidney axis in cerebral small vessel disease: Chronic kidney disease as an active mediator, not just a confounder. Hypertens. Res..

[91] Heo C.M., Yi J., Lee D.A., Park K.M., Lee Y.J., Park S., Kim Y.W., Ko J., Djojo A.Y., Park B.S. (2025). Analysis of functional brain connectivity in patient with end-stage kidney disease undergoing peritoneal dialysis using functional near infrared spectroscopy. PLoS ONE.

[92] Xu C., Miao H., Chen Y., Liao L. (2025). Crosstalk of kidney and brain in diabetes-related cognitive impairment and therapeutic strategies. Front. Endocrinol..

[93] Xie Z., Tong S., Chu X., Feng T., Geng M. (2022). Chronic Kidney Disease and Cognitive Impairment: The Kidney-Brain Axis. Kidney Dis..

[94] Yan Q., Liu M., Xie Y., Lin Y., Fu P., Pu Y., Wang B. (2024). Kidney-brain axis in the pathogenesis of cognitive impairment. Neurobiol. Dis..

[95] Zhou X., Sun Y., Yang G. (2025). 5-Methoxytryptophan improves cerebrovascular injury induced by chronic kidney disease through NF-kappaB pathway. In Vitro Cell. Dev. Biol. Anim..

[96] Sun X., Liu Y., Liu Y., Ai S., Luo P. (2025). Predictive Value of 5-Methoxytryptophan on Clinical Outcome in Patients with Sepsis Associated Acute Kidney Injury. J. Inflamm. Res..

[97] Jia Y., Shi Y., Wang J., Liu H., Wang H., Huang Y., Liu Y., Chen P., Peng J. (2025). Astragalin attenuates caerulein-induced acute pancreatitis by targeting the NLRP3 signaling pathway and gut microbiota. Bioresour. Bioprocess..

[98] Pan H., Yang S., Kulyar M.F., Ma H., Li K., Zhang L., Mo Q., Li J. (2025). Lactobacillus fermentum 016 Alleviates Mice Colitis by Modulating Oxidative Stress, Gut Microbiota, and Microbial Metabolism. Nutrients.

[99] Gong J., Lu H., Li Y., Xu Q., Ma Y., Lou A., Cui W., Song W., Qu P., Chen Z. (2025). ACE2 shedding exacerbates sepsis-induced gut leak via loss of microbial metabolite 5-methoxytryptophan. Microbiome.

[100] Zhou L., Wang X., Zhang Y., Xie Y., Cui R., Xia J., Sun Z. (2024). Renal Metabolomics Study and Critical Pathway Validation of Shenkang Injection in the Treatment of Chronic Renal Failure. Biol. Pharm. Bull..

[101] Hoffman R.M. (2019). Methionine Restriction and Life-Span Extension. Methods Mol. Biol..

[102] Green C.L., Trautman M.E., Chaiyakul K., Jain R., Alam Y.H., Babygirija R., Pak H.H., Sonsalla M.M., Calubag M.F., Yeh C.Y. (2023). Dietary restriction of isoleucine increases healthspan and lifespan of genetically heterogeneous mice. Cell Metab..

[103] Huang X., Zhang F., Yang Y., Liu J., Tan X., Zhou P., Tang X., Hu J., Chen L., Yuan M. (2025). Curcumin-copper complex nanoparticles as antioxidant nanozymes for acute kidney injury alleviation. Mater. Today Bio.

[104] Hu K., Wang P., Zhao Y., Zhang T., Zhao L., Su Y., Wen H., Liu C., Zou G., Wei L. (2025). Hypoxia-responsive modules via tunable hydrophobicity reversal enhance renal-targeted release of CD36-interfering nanoparticles to ameliorate acute kidney injury. J. Control. Release.

[105] Verhoven B., Tong Y., Chlebeck P., Zhong W., Zeng W., Jennings H., Miller B., Heise G., Levitsky M., Xie R. (2026). Attenuating Ischemia and Reperfusion Injury Using NAD+-Loaded Nanoparticles in Mouse Kidneys. Transplant. Direct.

[106] Wu J., Jin Z., Zheng H., Yan L.J. (2016). Sources and implications of NADH/NAD(+) redox imbalance in diabetes and its complications. Diabetes Metab. Syndr. Obes..

[107] Alaygut D., Ozturk I., Ulu S., Gungor O. (2023). NETosis and kidney disease: What do we know?. Int. Urol. Nephrol..

[108] Hammouri D., Weis T., Siskind L.J. (2025). Macrophage Plasticity and Functional Dynamics in Acute Kidney Injury and Its Progression to Chronic Kidney Disease. Semin. Nephrol..

[109] Valizadeh Gorji A., Fakhredini F., Soltanpour F., Mansouri E. (2025). Investigating the Effect of Secretome Secreted from Adult Rat Kidney-Derived Stem Cells on the Expression of miR375, miR494 and Tissue Changes in Glycerol-Induced Acute Kidney Injury. Adv. Biomed. Res..

[110] Abugomaa A., Elbadawy M. (2020). Olive leaf extract modulates glycerol-induced kidney and liver damage in rats. Environ. Sci. Pollut. Res. Int..

[111] Li R., Xia J., Shi C., Zhang K., Qu Y., He G., Fu Z., Deng L., Liu R., Wang X. (2025). Direct pharmacological targeting of Piezo1 by Paeoniflorin: A novel therapeutic approach for renal fibrosis. J. Adv. Res..

[112] Zhang X., Li S., Li S., Liu B., Ding G., Wu M., Zhang Y., Huang S., Gong W., Jia Z. (2025). Endothelial Lon protease 1 facilitates the redox balance to prevent glomerulosclerosis by acting on superoxide dismutase 2 ubiquitination. Redox Biol..

[113] Zhang Q., Wu G., Guo S., Liu Y., Liu Z. (2020). Effects of tristetraprolin on doxorubicin (adriamycin)-induced experimental kidney injury through inhibiting IL-13/STAT6 signal pathway. Am. J. Transl. Res..

[114] Kimura A., Ishida Y., Hayashi T., Wada T., Yokoyama H., Sugaya T., Mukaida N., Kondo T. (2006). Interferon-gamma plays protective roles in sodium arsenite-induced renal injury by up-regulating intrarenal multidrug resistance-associated protein 1 expression. Am. J. Pathol..

[115] Riaz M.A., Nisa Z.U., Mehmood A., Anjum M.S., Shahzad K. (2019). Metal-induced nephrotoxicity to diabetic and non-diabetic Wistar rats. Environ. Sci. Pollut. Res. Int..

[116] Yan L.-J., Allen D.C. (2021). Cadmium-Induced Kidney Injury: Oxidative Damage as a Unifying Mechanism. Biomolecules.

[117] Diwan V., Brown L., Gobe G.C. (2018). Adenine-induced chronic kidney disease in rats. Nephrology.

[118] Ho H.J., Kikuchi K., Oikawa D., Watanabe S., Kanemitsu Y., Saigusa D., Kujirai R., Ikeda-Ohtsubo W., Ichijo M., Akiyama Y. (2021). SGLT-1-specific inhibition ameliorates renal failure and alters the gut microbial community in mice with adenine-induced renal failure. Physiol. Rep..

[119] Liu H., Wang Y., Wang Y., Yan L.-J. (2024). Rodent Models of Streptozotocin-Induced Diabetes as Suitable Paradigms for Studying Diabetic Kidney Disease. Free Radic. Antioxid..

[120] Ikeda Y., Enomoto H., Tajima S., Izawa-Ishizawa Y., Kihira Y., Ishizawa K., Tomita S., Tsuchiya K., Tamaki T. (2013). Dietary iron restriction inhibits progression of diabetic nephropathy in db/db mice. Am. J. Physiol. Renal Physiol..

[121] Agil A., Chayah M., Visiedo L., Navarro-Alarcon M., Ferrer J.M.R., Tassi M., Reiter R.J., Fernandez-Vazquez G. (2020). Melatonin Improves Mitochondrial Dynamics and Function in the Kidney of Zucker Diabetic Fatty Rats. J. Clin. Med..

[122] Li C.Y., Ma W.X., Yan L.J. (2020). 5-Methoxyindole-2-Carboxylic Acid (MICA) Fails to Retard Development and Progression of Type II Diabetes in ZSF1 Diabetic Rats. React. Oxyg. Species.

[123] Zhai L., Gu J., Yang D., Hu W., Wang W., Ye S. (2016). Metformin ameliorates podocyte damage by restoring renal tissue nephrin expression in type 2 diabetic rats. J. Diabetes.

[124] Skovso S. (2014). Modeling type 2 diabetes in rats using high fat diet and streptozotocin. J. Diabetes Investig..

[125] Glastras S.J., Chen H., Teh R., McGrath R.T., Chen J., Pollock C.A., Wong M.G., Saad S. (2016). Mouse Models of Diabetes, Obesity and Related Kidney Disease. PLoS ONE.

[126] Wilson R.D., Islam M.S. (2012). Fructose-fed streptozotocin-injected rat: An alternative model for type 2 diabetes. Pharmacol. Rep..

[127] Pan X., Olatunji O.J., Basit A., Sripetthong S., Nalinbenjapun S., Ovatlarnporn C. (2024). Insights into the phytochemical profiling, antidiabetic and antioxidant potentials of Lepionurus sylvestris Blume extract in fructose/streptozotocin-induced diabetic rats. Front. Pharmacol..

[128] Zhou S., Ling X., Meng P., Liang Y., Shen K., Wu Q., Zhang Y., Chen Q., Chen S., Liu Y. (2021). Cannabinoid receptor 2 plays a central role in renal tubular mitochondrial dysfunction and kidney ageing. J. Cell. Mol. Med..

[129] Azman K.F., Zakaria R. (2019). D-Galactose-induced accelerated aging model: An overview. Biogerontology.

[130] Guo L., Wu P., Li Q., Feng Q., Lin X., Luo Y., Wang Y., Wu M., Cai F., Zhang J. (2025). NUAK1 Promotes Diabetic Kidney Disease by Accelerating Renal Tubular Senescence via the ROS/P53 Axis. Diabetes.

[131] Wang Q.L., Xing W., Yu C., Gao M., Deng L.T. (2021). ROCK1 regulates sepsis-induced acute kidney injury via TLR2-mediated endoplasmic reticulum stress/pyroptosis axis. Mol. Immunol..

[132] Arslan M., Kucuk A., Bozok U.G., Ergorun A.I., Sezen S.C., Yavuz A., Kavutcu M. (2023). The role of pomegranate seed oil on kidney and lung tissues in the treatment of sepsis: Animal pre-clinical research. J. Infect. Dev. Ctries..

[133] Wang X.H., Ao Q.G., Cheng Q.L. (2021). Caloric restriction inhibits renal artery ageing by reducing endothelin-1 expression. Ann. Transl. Med..

[134] Liu J.R., Cai G.Y., Ning Y.C., Wang J.C., Lv Y., Guo Y.N., Fu B., Hong Q., Sun X.F., Chen X.M. (2020). Caloric restriction alleviates aging-related fibrosis of kidney through downregulation of miR-21 in extracellular vesicles. Aging.

[135] Serna J.D.C., Amaral A.G., da Silva C.C.C., Munhoz A.C., Vilas-Boas E.A., Menezes-Filho S.L., Kowaltowski A.J. (2022). Regulation of kidney mitochondrial function by caloric restriction. Am. J. Physiol. Renal Physiol..

[136] Athinarayanan S.J., Roberts C.G.P., Vangala C., Shetty G.K., McKenzie A.L., Weimbs T., Volek J.S. (2024). The case for a ketogenic diet in the management of kidney disease. BMJ Open Diabetes Res. Care.

[137] Mikami D., Kobayashi M., Uwada J., Yazawa T., Kamiyama K., Nishimori K., Nishikawa Y., Morikawa Y., Yokoi S., Takahashi N. (2019). beta-Hydroxybutyrate, a ketone body, reduces the cytotoxic effect of cisplatin via activation of HDAC5 in human renal cortical epithelial cells. Life Sci..

[138] Liu H., Yan L.-J. (2023). The Role of Ketone Bodies in Various Animal Models of Kidney Disease. Endocrines.

[139] Yan L.J. (2021). Folic acid-induced animal model of kidney disease. Anim. Models Exp. Med..

